# The role of long non-coding RNA FGD5-AS1 in cancer

**DOI:** 10.1080/21655979.2022.2067292

**Published:** 2022-04-27

**Authors:** Na He, Linbiao Xiang, Lei Chen, Haobin Tong, Keshen Wang, Jie Zhao, Feixue Song, Hanteng Yang, Xinyuan Wei, Zuoyi Jiao

**Affiliations:** a Department of Oncology, Lanzhou University Second Hospital, Lanzhou, Gansu, China; b Department of General Surgery, Lanzhou University Second Hospital, Lanzhou, Gansu, China

**Keywords:** Long noncoding RNA, FGD5-AS1, human cancers, function, molecular mechanism

## Abstract

Long noncoding RNAs (lncRNAs) refers to a class of RNAs that have at least 200 nucleotides and do not encode proteins, and the relationship between lncRNA and cancer has recently attracted considerable research attention. The lncRNA FGD5-AS1 is a newly discovered lncRNA with a length of 3772 nucleotides. Studies have found that FGD5-AS1 is abnormally highly expressed in many cancer tissues and was closely related to the lymph node metastasis, tumor invasion, survival time, and recurrence rate of various cancers. Mechanistic analyses show that FGD5-AS1 can stabilize mRNA expression by sponging miRNA, which not only induces cancer cell proliferation, metastasis, invasion, and chemoresistance in vitro, but also promotes tumor growth and metastasis in vivo. In addition, FGD5-AS1 can serve as a diagnostic or prognostic marker for a variety of cancers. This review demonstrates the clinical significance of FGD5-AS1 in human cancer and its role in tumorigenesis and tumor progression.

## Graphical abstract

**Figure:**
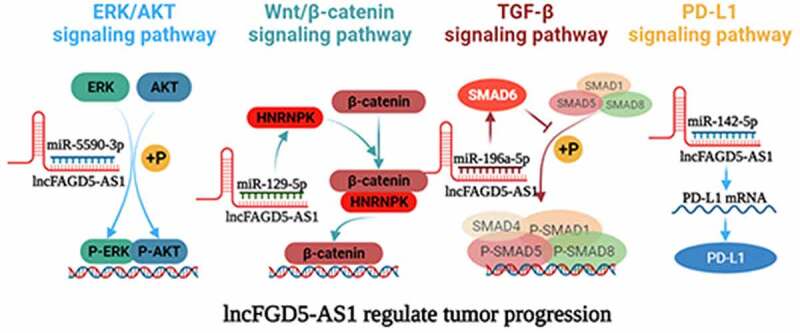

## Highlights

- We summarized and reviewed the reports on FGD5 - AS1 in recent years, sorted out its abnormal expression and physiological significance in different cancers.
- We introduce FGD5-AS1 involved regulatory networks and signaling pathways in detail, and prospected the future work.
- This review may help to the development of new biomarkers and therapeutic avenues to improve cancer outcomes.

## Introduction

Long noncoding RNAs are a class of transcripts longer than 200 nucleotides without promoters and terminators; therefore, they do not have protein-coding functions [1,2]. With the rapid development of gene sequencing technology, a large number of lncRNAs have been identified. Initially, lncRNAs were mistaken for gene transcription noise without functions; therefore, they attracted limited attention. However, further research showed that lncRNAs regulate chromatin remodeling, DNA methylation, histone modification, and RNA metabolism [3]. Mature lncRNAs can interact with a variety of molecules to form supramolecular structures, such as RNA-RNA, RNA-DNA, RNA-protein, DNA-RNA-protein, or DNA-RNA-RNA complexes [4]. Once transcribed, lncRNAs exert cis-control over local gene expression or trans-control over remote gene expression, leading to tissue-specific gene silencing or activation, thus acting as oncogenes or tumor suppressor genes [5,6]. Report have shown that lncRNAs is abnormally expressed in gastric cancer, breast cancer, hepatocellular carcinoma, and other cancer tissues [7,8], and their level of expression is significantly related to tumor growth [9], metastasis [10], chemoresistance [11], angiogenesis [12], tumor metabolism [13], and cancer stem cells [14]. Therefore, the regulatory mechanisms of lncRNAs in the pathological process of cancer must be better understood to promote more targeted clinical treatments.

FYVE RhoGEF and PH domain containing 5(FGD5) belong to the Rho guanine nucleotide exchange factor (Rho GEF) family [15,16] and have been demonstrated to promote angiogenesis by increasing VEGF expression in endothelial cells [17,18]. At present, few studies have focused on FGD5 in oncology, although the function of its antisense RNA (FGD5-AS1) in various tumors has attracted much attention. FGD5-AS1 is a novel lncRNA with a length of 3772 nucleotides located on chromosome 3, and it was first reported in 2018 as being involved in the lncRNA-associated ceRNA network that mediates reduced inflammation in periodontitis [19], and subsequently proven to be associated with the poor prognosis of renal clear cell carcinoma [20]. Overexpression of FGD5-AS1 has been identified in more malignant cancers, and deletion of FGD5-AS1 significantly reduces the migration, invasion, stemness, immune microenvironment, and proliferation of various tumor cells [21–24]. In this review, we summarized current studies on FGD5-AS1 and its underlying the mechanisms of which in numerous cancers. Moreover, the relation between the abnormal expression of FGD5-AS1 and clinical characteristics and the influence of such expression on multiple biological functions of cancers are also discussed. The findings indicated that FGD5-AS1 may serve as a multitumor biomarker or therapeutic target.

## Abnormal expression and clinical significance of FGD5-AS1 in various cancer types

In recent years, numerous studies have shown that FGD5-AS1 is abnormally expressed in various cancers, and this alteration is closely associated with the malignant performance of tumors and the poor prognosis of patients (Table 1). Moreover, studies have confirmed that lncRNAs serve as competitive endogenous RNAs (ceRNAs) to inhibit the expression of messenger RNAs (mRNAs) by sponging miRNAs [25]. As shown in Table 2, 17 miRNAs have been found to participate in the ceRNA network of FGD5-AS1. In addition, these miRNAs have at least 22 targeted mRNAs that directly or indirectly participate in the biological behavior of tumor cells, such as epithelial-mesenchymal transition (EMT), proliferation, invasion, metastasis, chemoresistance, and apoptosis. We summarize the biological functions and the complex mechanisms of FGD5-AS1 in various cancers.

**Table 1.** The significance of FGD5-AS1 dysregulated in clinicopathological characteristics, chemoresistance, and prognosis of various cancers

Cancer type	Expression	Sample	Related clinicopathological features	Prognostic implication of FGD5-AS1 overexpression	Property	PMID	Ref.
Renal cell carcinoma	Upregulation	28 tissue samples from RC patients	Lymph node metastasis, distant metastasis	Poor	Oncogene	32,964,964	^[57]^
Kidney renal clear cell carcinoma	Downregulation	539 tissue samples from ccRCC and 72 adjacent normal renal tissues	Tumor stage, histological grade, TNM stage, VHL mutant	Favorable	Tumor suppressor	33,854,630	^[24]^
Gastric cancer	Upregulation	45 pairs of GC tissues and adjacent normal tissues	-	-	Oncogene	2,849,774	^[30]^
	Downregulation	30 pairs of GC tissues and adjacent normal tissues	Tumor stage, TNM stage, Lymph node metastasis, distant metastasis, serous membrane infiltration	Favorable	Tumor suppressor	33,892,661	^[32]^
Hepatocellular carcinoma	Upregulation	22 pairs of HCC tissues and adjacent normal tissues	-	Poor	Oncogene	32,257,949	^[34]^
	Upregulation	32 cisplatin-resistance and 28 cisplatin-sensitive HCC patients	Cisplatin resistance	-	Oncogene	34,519,634	^[35]^
Non-small cell lung cancer	Upregulation	50 pairs of NSCLC tissues and adjacent normal tissues	TNM stage, tumor size	Poor	Oncogene	31,919,528	^[40]^
	Upregulation	30 cisplatin-resistance and 30 cisplatin-sensitive NSCLC patients	Cisplatin resistance	-	Oncogene	32,534,055	^[42]^
	Upregulation	35 cisplatin-resistance and 30 cisplatin-sensitive NSCLC patients	Tumor size, lymph node metastasis	Poor	Oncogene	33,550,957	^[39]^.
	Upregulation	65 tissue samples from NSCLC patients	TNM stage, distant metastasis	-	Oncogene	33,550,920	^[41]^
	Upregulation	25 cisplatin-resistance and 21 cisplatin-sensitive NSCLC patients	Cisplatin resistance	-	Oncogene	33,416,094	^[21]^
Colorectal cancer	Upregulation	40 5-fu resistance and 40 5-fu sensitive CRC patients	5-fu resistance	-	Oncogene	34,589,581	^[22]^
Glioblastoma	Upregulation	30 pairs of GMB tissues and adjacent normal tissues	-	-	Oncogene	32,848,452	^[64]^
	Upregulation	64 tissue samples from GMB and 35 adjacent normal tissues	-	Poor	Oncogene	32,585,241	^[62]^
Osteosarcoma	Upregulation	94 tissue samples from GMB and 100 adjacent normal tissues	Tumor size, TNM stage	Poor	Oncogene	33,408,528	^[69]^
	Upregulation	50 pairs of OS tissues and adjacent normal tissues	TNM stage, differentiation	Poor	Oncogene	33,891,267	^[70]^
Breast cancer	Upregulation	23 pairs of BC tissues and adjacent normal tissues	-	-	Oncogene	33,880,593	^[45]^
	Upregulation	50 pairs of BC tissues and adjacent normal tissues	Radio-response, differentiation, TNM stage, lymph node metastasis	Poor	Oncogene	34,221,989	^[23]^
Ovarian cancer	Upregulation	52 pairs of ovarian cancer tissues and adjacent normal tissues	Lymph node metastasis, tumor stage	Poor	Oncogene	33,974,163	^[56]^
Oral cancer	Upregulation	30 pairs of oral cancer tissues and adjacent normal tissues	Tumor size, TNM stage	-	Oncogene	31,899,825	^[29]^
Laryngeal squamous cell carcinoma	Upregulation	38 pairs of LSCC cancer tissues and adjacent normal tissues	TNM stage, lymph node metastasis	-	Oncogene	34,003,510	^[71]^
Melanoma	Upregulation	188 pairs of melanoma tissues and adjacent normal tissues	Tumor size, TNM stage	Poor	Oncogene	32,997,827	^[72]^

**Table 2.** Functions and mechanisms of FGD5-AS1 in different cancer cell lines

Cancer type	Cell lines	Expression level	Effect in vitro	Effect in vivo	miRNA	Protein	Pathway	PMID	Ref.
Renal cell carcinoma	86-O, ACHN, SN12PM6, HK-2	Upregulation	Metastasis**↑**, invasion**↑**, proliferation**↑**, EMT**↑**	-	miR-5590-3p		ERK/ AKT	32964964	^[57]^
Kidney renal clear cell carcinoma	OSRC2, ACHN, A498, 769-P, 786-O	Downregulation	-	-	-	-	-	33,854,630	^[24]^
Gastric cancer	AGS, NIC-N87, SNU-1, SNU5, SNU-16, MKN-45, SGC-7901, BGC-823, MKN28, GES-1	Upregulation	Proliferation**↑**, 5-Fu resistance**↑**	Tumor growth**↑**	miR-153-3p	CITED2	-	2,849,774	^[30]^
	MKN74, MKN45, 293 T	Downregulation	Metastasis↓, invasion↓, proliferation↓	-	miR-196a-5p	SMAD6	TGF-β	33,892,661	^[32]^
Hepatocellular carcinoma	Huh7, Hep3B	Upregulation	Metastasis**↑**, invasion**↑**, proliferation↑, cisplatin resistance**↑**, clone formation**↑**	-	miR-153-3p	TWF1	-	34,519,634	^[35]^
Non-small cell lung cancer	IMR-90, NCI-H1703, NCI-H1793, NCI-H1869	Upregulation	Metastasis↑, invasion↑, proliferation↑, clone formation↑,	-	miR-107	FGFRL1	-	31,919,528	^[40]^
	A549, H1299	Upregulation	Proliferation↑, cisplatin resistance**↑**	-	miR-140-5p	WEE1	-	32,534,055	^[42]^
	H1650, H1299, SPC-A-1, A549, BEAS-2B	Upregulation	Proliferation**↑**, invasion↑, EMT**↑**	Lung metastasis**↑**	miR-493-5p	DDX5	-	33,550,957	^[39]^
	H358, H1299, PC-9, A549, BEAS-2B	Upregulation	Metastasis**↑**, invasion**↑**, proliferation**↑**		miR-944	MACC1	-	33,550,920	^[41]^
	A549, HCC827	Upregulation	Metastasis**↑**, invasion**↑**, proliferation↑, cisplatin resistance**↑**	-	miR‑142‑5p	PD‑L1	-	33,416,094	^[21]^
Colorectal cancer	HT29, LoVo, SW480, SW620, HCT116, HcoEpic	Upregulation	Metastasis**↑**, invasion**↑**, proliferation**↑**	-	miR-302e	CDCA7	-	31,332,696	^[37]^
	LoVo, HCT-116, HT-29, DLD-1	Upregulation	5-Fu resistant**↑**	5-Fu resistant**↑**	miR-330-3p	HK2	-	34,589,581	^[22]^
Glioblastoma	A172, U87, U251, T98G, LN229, NHA	Upregulation	Metastasis**↑**, invasion**↑**, proliferation**↑**	-	miR-103a-3p	TPD52	-	32,848,452	^[64]^
	A172, U251MG, SHN44, LN229	Upregulation	Metastasis↑, invasion↑, proliferation↑, clone formation↑,	Tumor growth**↑**	miR-129-5p	HNRNPK	Wnt/β-catenin	32,585,241	^[62]^
	HA, U251, SHG139, U87	Upregulation	Metastasis↑, invasion↑, proliferation↑, clone formation↑,	Tumor growth**↑**	-	β-catenin, cyclinD1	Wnt/β-catenin	32,801,867	^[63]^
Osteosarcoma	U2OS, HOS, SaOS-2, SW1353, hFOB1.19	Upregulation	Metastasis↑, invasion↑, proliferation↑, apoptosis↓	-	miR-320b	-	-	33,408,528	^[69]^
	143B, HOS, U2OS, MG63, hFOB1.19	Upregulation	Metastasis↑, invasion↑, proliferation↑	-	miR‑506‑3p	RAB3D	-	33,891,267	^[70]^
Breast cancer	MCF‑7, MDA‑MB‑231, MDA‑MB‑468, SKBR3	Upregulation	Metastasis↑, invasion↑, proliferation↑		miR‑195‑5p	NUAK2	-	33,880,593	^[45]^
	MCF-7, MDA-MB231	Upregulation	clone formation↑, cell cycle arrest↓, apoptosis↓, radioresistance↑		miR-497-5p	MACC1	-	34,221,989	^[23]^
Cervical Cancer	HeLa, SiHa, C33A, CasKi, H8	Upregulation	Metastasis↑, invasion↑, M2 macrophages↑	-	miR-129-5p	BST2	-	34,692,852	^[53]^
Ovarian cancer	OVCAR3, SKOV3, HO8910, A2780, COV644, IOSE	Upregulation	Metastasis↑, invasion↑, proliferation↑	Lung metastasis**↑**	miR‑142‑5p	PD-L1	-	33,974,163	^[56]^
Oral cancer	SCC4, SCC-25, CAL-27	Upregulation	Metastasis↑, invasion↑, proliferation↑	Proliferation↑	miR-153-3p	MCL1	-	32,675,387	^[28]^
	HOK, HSC-4, SCC25, CAL-27, SCC-9	Upregulation	Metastasis↑, invasion↑, proliferation↑ apoptosis↓	Proliferation↑	miR-520b	USP21	-	31,899,825	^[29]^
Laryngeal squamous cell carcinoma	Hep2, TU212, TU686, SCC‐2, 16HBE	Upregulation	Metastasis↑, invasion↑, proliferation↑ apoptosis↓	-	miR‐497‐5p	SEPT2	-	34,003,510	^[71]^
pancreatic adenocarcinoma	Capan-1, BxPc3, PANC-1, SW1990, HPDE-C7	Upregulation	Metastasis↑, invasion↑, proliferation↑ apoptosis↓, clone formation↑,	-	miR-520a-3p	KIAA1522	-	33,794,727	^[73]^

## Digestive system

### Oral cance

As the sixth most malignant tumor worldwide, oral cancer (OC) is prone to regional invasion and lymph node metastasis, and the prognosis is extremely poor with a 5-year survival rate of less than 50% [26,27]. Therefore, early monitoring and detection of OC are important. It has been verified that FGD5-AS1 is highly expressed in OC tissues and cells compared with adjacent and normal human oral epithelial cells, whereas knockdown of FGD5-AS1 can significantly inhibit the vitality, migration, and invasion of OC cells [28]. In addition, further functional assays showed that FGD5-AS1 promotes OC progression through the miR153-3p/MCL1 axis, and overexpression of miR153-3p or inhibition of MCL1 could reverse the tumor-promoting effect of FGD5-AS1 [28]. Liu et al. [29] also reported that FGD5-AS1 could promote OSCC progression, and they confirmed via luciferase reported assays that FGD5-AS1 sponges miR-520b and restores the expression of USP21, which could promote OSCC cell growth and migration and inhibit OSCC cell apoptosis [29]. In summary, increasing information about FGD5-AS1 indicated that it might be a new target for oral cancer treatment.

### Gastric cancer

A previous report demonstrated that FGD5-AS1 is aberrantly overexpressed in gastric cancer (GC) tissues compared with adjacent normal tissues [30]. Based on the downstream Hsa-miR-1533p/CITED2 signaling axis, FGD5-AS1 upregulation could markedly promote GC cell proliferation, chemoresistance, and in vivo tumorigenicity [30,31]. Interestingly, another report showed that FGD5-AS1 plays a tumor suppressor role in GC ny inhibiting the invasion, metastasis, and proliferation of GC both in vitro and in vivo though the regulation of the miR-196a-5p/SMAD6/BMP axis [32]. Unfortunately, the conclusions of these two studies are inconsistent; thus, the role of FGD5-AS1 in GC needs to be further determined.

### Hepatocellular carcinoma

Most patients with hepatocellular carcinoma(HCC) are already in the advanced stage of diagnosis, and even after surgery, they still have a high risk of recurrence and metastasis, resulting in poor prognosis [33]. Therefore, to determine the underlying mechanism of HCC and identify biomarkers for early diagnosis and prognostic prediction, Zhang et al. [34] constructed an ‘mRNA-miRNA-lncRNA’ triple subnetwork based on multiple databases. As one of the predicted lncRNA clusters, FGD5-AS1 was significantly upregulated and linked to the poor prognosis in patients with HCC [34]. In addition, silencing of FGD5-AS1 significantly restrained the HCC cell viability and invasion and facilitated apoptosis [35]. FGD5-AS1 was also shown to upregulate twinfilin actin binding protein 1 (TWF1) expression by sponging microRNA-153-3P, and then promote cisplatin resistance in HCC cells [35]. These results illustrated that FGD-AS1 could be a therapeutic target for HCC; however, verifying the role of FGD-AS1 as a predictive biomarker requires large-sample clinical data for further validation.

### Colorectal cancer

Colorectal cancer(CRC) remains the third most common malignancy and the second leading cause of cancer-related mortality worldwide [36]. Therefore, effective therapeutic targets must be identified for CRC. Li et al. [37] and Gao et al. [22] reported that FGD5-AS1 is highly ectopically expressed in CRC cell lines and tissues, and functional assays indicated that FGD5-AS1 promoted metastasis and invasion, suppressed apoptosis, and facilitated CRC cell proliferation by upregulating the cell division cycle associated 7 (CDC7), which can start DNA replication, inhibit apoptosis and act as an oncogene in various tumors [38]. Moreover, both in vitro and in vivo trials revealed that inhibition of FGD5-AS1 can release the inhibition of tumor suppressor miR-330-3p, thereby down-regulating the expression of oncogene HK2 and the level of glycolysis and overcoming EGFR-induced 5-fu resistance in rectal cancer cells. On the other hand, EGFR inhibitor can significantly down-regulate the expression of FGD5-AS1, which makes the combination of EGFR inhibitor and 5-FU show better anti-tumor effect [22]. These results suggest that the FGD5-AS1 may be a novel therapeutic target for CRC.

## Respiratory system

### Lung cancer

Previous studies have proven that FGD5-AS1 exhibits a cancer-promoting role in non-small-cell lung cancer (NSCLC). The expression of FGD5-AS1 in NSCLC tissues and cells is relatively higher than that in adjacent tissues and normal cells, and its high expression in NSCLC tissues is significantly related to poor pathological indicators, including a large tumor diameter and high lymph node positive rate [39]. In cell experiments, FGD5-AS1 could significantly promoted NSCLC cell proliferation, metastasis, invasion, clone formation, and macrophage M2 polarization and inhibited NSCLC cell apoptosis [39–41]. In addition, FGD5-AS1 silencing completely reversed the above phenotypes. FGD5-AS1 upregulation contributed to cisplatin resistance both in vitro and in vivo by upregulating programmed cell death 1-ligand 1(PD1-L1) and fibroblast growth factor receptor like 1 (FGFRL1) [21,42]. These findings may provide a potential therapeutic target for NSCLC; however, the regulatory mechanism of FGD5-AS1 also needs to be further explored.

## Genital system

### Breast cancer

From the report of 2021 cancer statistics, breast cancer (BC) has become the most common malignancy among women worldwide with more than 275,000 new cases diagnosed annually [43]. Although considerable progress has been made in endocrine therapy and Her-2 targeted therapy in recent years, triple negative breast cancer (TNBC) still lacks satisfactory targeted therapy [44]. As such, there is a critical need to find new molecular targets for breast cancer therapy. FGD5-AS1 is significantly overexpressed in breast cancer tissues and cells and could promote the proliferation, glycolysis, migration, and invasion of BC cells by binding with miR-195-5p and removing the inhibitory effect on NUAK2 expression; moreover, silencing FGD5-AS1 leads to the opposite phenotype [45]. Li et al. [23] also confirmed the above results and found that FGD5-AS1 can competitively bind with miR-497-5p, which promotes the expression of MACC1 and the radiotherapy tolerance of BC cells. Furthermore, FGD5-AS1 deficiency could promote breast cancer cell apoptosis and sensitize the cells to X-rays by activating the expression of BAX, Caspase 3, and Caspase 9 [23]. These results indicate that FGD5-AS1 plays an important role in the progression of BC. However, further research is required before its clinical application.

### Cervical cancer

According to cancer statistics, cervical cancer (CC) is one of the most common gynecologic malignancies worldwide and causes more than 570000 new cases and 311,000 deaths per year [46].Human papillomavirus (HPV) infection, premature or bad new life, and smoking are risk factors for cervical cancer [47]. In addition to radiotherapy, chemotherapy, and surgery, immunotherapy has gradually attracted much attention. Moreover, because most cervical cancer patients have HPV infection, the role of the immune system in the pathogenesis and treatment of CC is particularly important [48–50]. Macrophages are immune cells that play an important role in the tumor microenvironment [51]. Macrophages can be divided based on their different functional characteristics into the antitumor classical activation type (M1 type) or protumor alternative activation type (M2 type), which can promote the inflammatory response and tumor progression [52]. Liu et al. [53] first reported that FGD5-AS1 was highly expressed in CC cells and promoted the proliferation, metastasis, and invasion of CC cells, they also found that FGD5-AS1 could upregulate the expression of bone marrow stromal cell antigen 2 (BST2) and thus promote M2 macrophage polarization by directly sponging miR-129-5p. In contrast, silencing FGD5-AS1 could significantly inhibit the polarization of M2 macrophages and the malignant phenotype of CC cells [53]. FGD5-AS1 seems to be a valuable therapeutic target for CC. However, the above study on FGD5-AS1 in CC lacks data support from animal experiments, and the mechanism needs to be further verified in future research.

### Ovarian cancer

Despite the continuous breakthroughs made in the treatment of cancer over the past decade, early diagnosis and screening strategies for ovarian cancer (OC) are still difficult to achieve [54]. More than two-thirds of patients with OC are at stage III and IV at the time of initial diagnosis, and they present a 5-year survival rate less than 25% [54]. Zhao et al. [55] used the TCGA database to analyze all lncRNA-miRNA-mRNA competing triplets related to the prognosis of OC, and the results showed that FGD5-AS1 was significantly associated with the prognosis of OC patients in each pathological stage according to a univariate Cox-PH analysis and an age-adjusted multivariate Cox-PH analysis [55]. Subsequently, FGD5-AS1 was found to be overexpressed in OC cells and tissues based on the high T staging, lymph node metastasis, and poor prognosis of patients with OC. Further gain-of-function and loss-of-function assays showed that FGD5-AS1 upregulated the expression of wild type PD-L1 by targeting miR-142-5P, thereby introducing tumor cells to escape immunity by suppressing the immune response and promoting the proliferation, metastasis and invasion of OC cells [56]. Therefore, FGD5-AS1 might serve as a new biomarker and therapeutic target for the diagnosis and treatment of OC.

## Urinary system

### Renal cancer

In renal cell carcinoma(RCC), the expression of FGD5-AS1 is significantly higher in cancer tissues than adjacent tissues, and FGD5-AS1 is also overexpressed in patients with metastasis compared with patients without metastasis [57]. Silencing FGD5-AS1 can significantly reduce the invasion and metastasis of RCC cells by negatively regulating miR-5590-3p [57]. However, FGD5-AS1 plays the opposite role in kidney renal clear cell carcinoma (KIRC). Zhu et al. [20] reconstructed a ceRNA network to determine the potential prognostic biomarkers in KIRC and pointed out that lncFGD5-AS1 could serve as a potential prognostic biomarker. Contrary to the finding for RCC, KIRC patients with high expression of FGD5-AS1 had a better prognosis [20]. In addition, it has been reported that the expression of FGD5-AS1 in KIRC is significantly lower than that in adjacent tissues and is negatively correlated to the tumor stage, metastasis, pathological stage, and histological grade [24]. It is well known that Hippel-Lindau(VHL) play a critical tumor suppressor role in KIRC, and VHL gene inactivation is by far the most common carcinogenic driving event in KIRC. Above studies also found that FGD5-AS1 and VHL were co-expressed and their expression levels were positively correlated [24]. . Unfortunately, there is no relevant basic research to further clarify this relationship. Eventually, FGD5-AS1 can be used as a valuable diagnostic and predictive biomarker, although it plays an opposite role in the progression of RCC and KIRC. Additional experiments and clinical trials are still needed to explain this difference.

## Nervous system

### Glioblastoma

Glioblastoma (GMB) is one of the most common malignant tumors in the central nervous system and accounts for approximately half of the primary intracranial tumors [58]. Due to its heterogeneity and high recurrence rate, the mortality of GBM is among the highest for nervous system [59,60]. Lin et al. [61] found that FGD5-AS1 is highly expressed in GMB tissues and was related to poor prognosis. Moreover, FGD5-AS1 has been shown to activate the Wnt/β-catenin pathway by mediating the miR-129-5p/HNRNPK axis, thereby promoting the proliferation and metastasis of GMB cells [62,63]. Su et al. [64] reported that TPD52 level is positively correlated with FGD5-AS1 expression. FGD5-AS1could regulate the expression of TPD52 through miR-103a-3p and knockout of FGD5-AS1 could significantly inhibit the growth of the GMB xenograft model in vivo as well as other malignant biological phenotypes. In summary, these findings further elucidate the mechanisms of FGD5-AS1 in the progression of GBM and provide a promising therapeutic target for GBM patients.

## Motor system

### Osteosarcoma

Osteosarcoma, which is also known as osteogenic sarcoma, is one of the most common malignant tumors in bone, and it is common in children and adolescents. Since osteosarcoma progresses rapidly and is frequently prone to bone metastasis and lung metastasis, the five-year survival rate of osteosarcoma is less than 30% [65–67]. lncRNAs have been demonstrated to participate in the development of osteosarcoma through epigenetic, transcriptional control, and posttranscriptional modifications [68]. As a new lncRNA, FGD5-AS1 is found to be highly expressed in the serum of osteosarcoma patients compared to normal controls and can be used as a biomarker for the diagnosis of osteosarcoma [69]. Consistent with its expression in serum, FGD5-AS1 is also highly expressed in osteosarcoma tissues and promotes osteosarcoma cell viability, invasion, and epithelial-mesenchymal transition (EMT) ability via specific binding to miR-320b [69]. Li et al. [70] also reported that FGD5-AS1 contains the binding site of the miR-506-3p seed sequence, which can negatively regulate the expression of miR-506-3p and weaken the inhibitory effect of miR-506-3p on the mRNA and protein expression of RAB3D, thereby promoting its high expression and the progression of osteosarcoma. FGD5-AS1 plays a promoting role in the pathogenesis of osteosarcoma and is expected to become a new target for osteosarcoma treatment.

### Other cancers

Reportes have indicated that FGD5‐AS1 overexpression increases cisplatin resistance in laryngeal squamous cell carcinoma (LSCC) by sponging miR‐497‐5p and upregulating septin 2 (SEPT2) [71]. Gao et al. [72] reported that high expression of FGD5-AS1 is associated with a larger tumor thickness, later tumor stage, and higher mortality of melanoma. Moreover, FGD5-AS1 is an independent factor for the overall survival and disease-free survival of patients with melanoma. In pancreatic adenocarcinoma (PAAD), FGD5-AS1 also functions as an oncogene and thus can expedite the proliferation, metastasis, and invasion of PAAD cells. Silencing FGD5-AS1 can reverse the inhibitory effect of miR-520a-3p on KIAA1522, thereby attenuating the progression of PAAD [73]. Consequently, FGD5-AS1 is a promising prognostic and therapeutic target for LSCC, PC, and melanoma.

### Bioinformatics analysis

**Figure 1.**
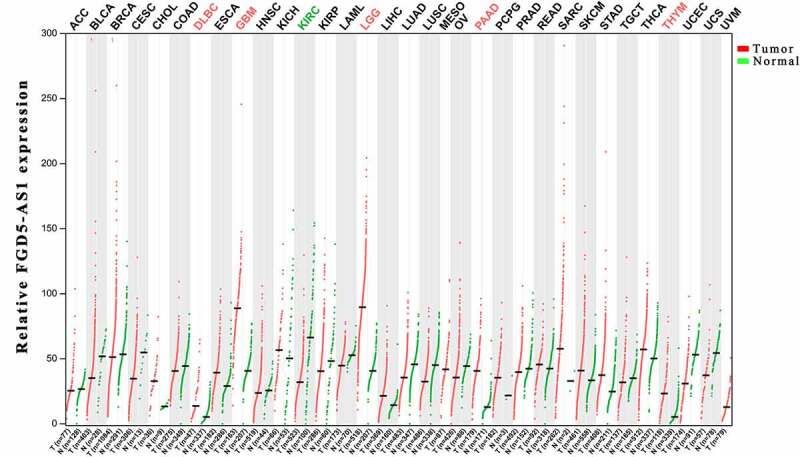
Expression of FGD5-AS1 in different cancer types.

**Figure 2.**
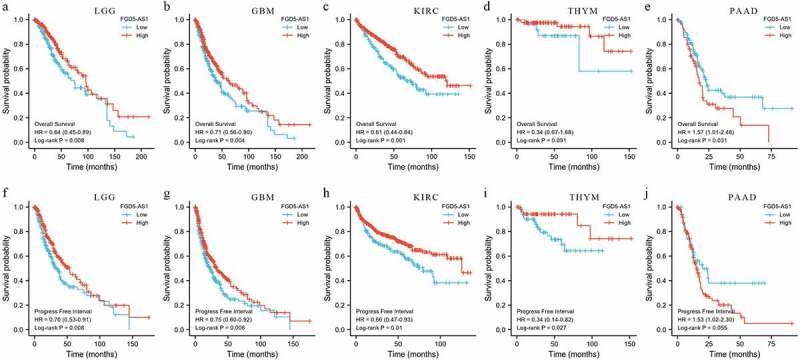
The prognostic roles of FGD5-AS1 in different cancer types. (A-E) Lowely expressed FGD5-AS1 had poor overall survival (OS) in patients with LGG, GBM, PAAD, and THYM and highly expression FGD5-AS1 had poor OS in patients with PAAD; (F-J) Lowely expressed FGD5-AS1 had poor Progress free interval (PFI) in patients with LGG, GBM, PAAD, and THYM and highly expression FGD5-AS1 had poor PFI in patients with PAAD.

In order to further clarify the expression profile of FGD5 – AS1 in recurrent carcinoma, we explored the expression and prognosis of FGD5-AS1 through the GEPIA website based on The Cancer Genome Atlas (TCGA) database [74]. The results showed that FGD5-AS1 was highly expressed in Brain low-grade glioma (LGG), GBM, PAAD and thymic carcinoma (THYM), and we also observed low expression of FGD5-AS1 in KIRC, which is consistent with the above finding (Figure 1) [20]. Meanwhile, we also investigated whether the differential expression of FGD5-AS1 was associated with the prognosis of cancer patients. Unexpectedly, although FGD5-AS1 is overexpressed in LGG, GBM, PAAD and THYM tumor tissues compared to para-cancer tissuess, high expression of FGD5-AS1 is related to better OS and PFS of LGG. We also observed the same results in GBM, KIRC and THYM. Besides, the high expression of FGD5-AS1 in PAAD is related to poor prognosis (Figure 2). In summary, FGD5 – AS1 can be used as a new marker for the diagnosis and prognosis of different cancers.

## FGD5-AS1 related signaling pathways

**Figure 3.**
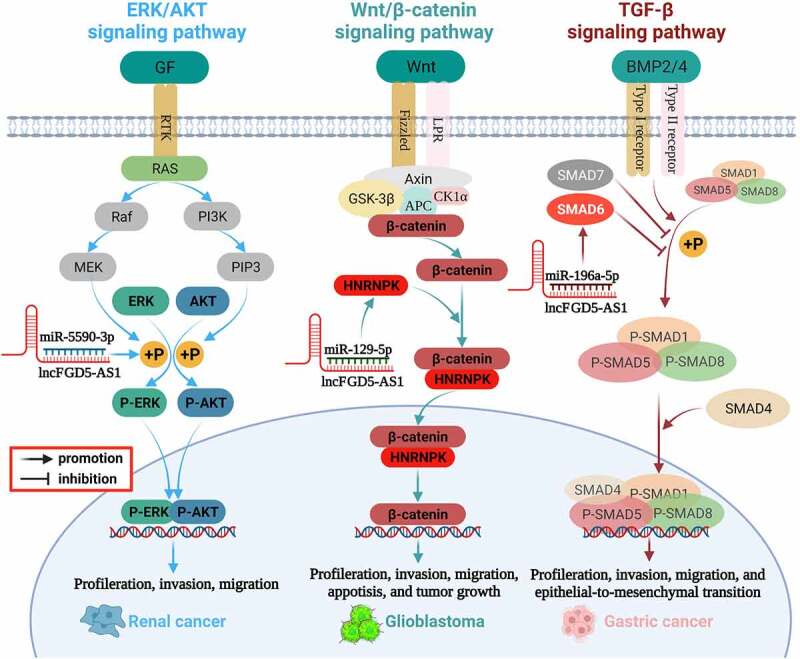
Mechanism of FGD5-AS1 in various cancer.

Previous studies have found that FGD5-AS1 is involved in the regulation of the TGF-β signaling pathway [32], ERK/AKT signaling pathway [57], and Wnt/β-catenin signaling pathway [62,63]. We summarize these signaling pathways and illustrated them in Table 2 and Figure 3.

### TGF-β signaling pathway

The transforming growth factor-β (TGF-β) family is widely distributed in various organisms and plays an important regulatory role in cell proliferation, cell differentiation, cell apoptosis, embryonic development, organ formation, immune function, inflammatory response, and wound repair [75]. Members of the TGF-β family include TGF-betas, activins, bone morphogenetic proteins (BMPS), growth, differentiation factors (GDFs), and Mullerian-inhibiting substance (MIS). Studies have revealed that BMPs can activate SMAD1/5/8 phosphorylation and promote SMAD1/5/8 expression by binding with BMP receptor proteins (BMPRS), thus participating in the pivotal process of EMT [76,77]. As one of the inhibitory Smads in the TGF-β signaling pathway, SMAD6 preferentially inhibits SMAD1/5/8 phosphorylation through the BMP type-I receptors ALK-3 and ALK-6 [77–79]. FGD5-AS1 can be used as a sponge of miR-196a-5p to upregulate SMAD6, inhibit SMAD1/5/8 phosphorylation, and participate in the TGF-β signaling pathway, thereby inducing the role of the BMP axis in the EMT process of gastric cancer cells [32].

### ERK/AKT signaling pathway

The mitogen-activated protein kinase (MAPK)/extracellular-signal regulated kinase (ERK) and phosphoinositide-3 kinase (PI3K)/Akt signaling networks are essential for the regulation of various cellular physiological behaviors. Phosphorylation is the main activation form of ERK and AKT, and abnormally activated ERK/AKT signaling is related to the proliferation, metastasis, invasion, and apoptosis of various cancers [80–83]. It has been reported that ERK/AKT is also a key pathway that regulates the invasion and progression of RCC, whereas inhibition of ERK and AKT phosphorylation can significantly inhibit the malignant phenotype of RCC cells [84,85]. FGD5-AS1 could competitively interact with miR-5590-3p and promote downstream ERK/AKT phosphorylation, thereby facilitating the proliferation and EMT of RCC cells [57].

### Wnt/β-catenin signaling pathway

The Wnt/β-catenin pathway is an evolutionarily conserved signaling pathway with an important role in regulating embryonic speech, tissue growth, stem cell homeostasis, and tissue regeneration [86–89]. Clinical and experimental data have shown that the abnormally activated Wnt/β-catenin signaling pathway is related to the occurrence and development of various cancers, and its inhibition induces an effective antitumor response [90,91]. Zhao found that the Wnt/β-catenin pathway is abnormally activated in glioma, which is significantly related to the high expression of FGD5-AS1 [63]. Furthermore, the expression of β-catenin and cyclin D1, the key components of the Wnt pathway, significantly decrease after FGD5-AS1 inhibition [63]. Further mechanistic studies revealed that FGD5-AS1 upregulates the expression of heterogeneous nuclear ribonucleoprotein K(HNPNPK) by sponging miR-129-5p, thereby activating Wnt/β-catenin signaling and promoting the malignant phenotype of GBM. Knockdown of FGD5-AS1 reduces the extranuclear accumulation of β-catenin, although this effect could be distinctly abolished by downregulation of miR-129-5p or overexpression of HNPNPK [62]. In addition, the suppressive effect of FGD5 silencing on GBM proliferation and invasion could be eliminated in cells by treatment with the Wnt/β-catenin pathway activator LiCl [62]. In summary, FGD5-AS1 is an important upstream activator of the Wnt/β-catenin signaling pathway that affects the progression of GBM.

### PD1/PD-L1 signaling pathway

As an important immunosuppressive factor, PD-L1 is a predictive biomarker for cancer immunotherapy and plays a crucial role in the malignant progression of different cancers, including but not limited to MM, OC, RCC, BC, as well as NSCLC [92–94]. The combination of PD-L1 and PD-1 inhibits T cell proliferation, IL-2 and IFN-γ secretion, inhibition of tumor-specific CD8^+^ T cell cytolytic activity, and induction of cytotoxic T lymphocyte apoptosis, induction and maintenance of T cell inactivation,, thus making TH2 dominant in tumor microenvironment and resulting in tumor immune escape [95]. It had been found that knockdown of FGD5-AS1 in NSCLC cells increased the ratio of CD8^+^ T cells and reduced the apoptosis of CD8^+^ T cells, which were completely reversed by blocking the PD1/PD-L1 interaction using the anti-PD-1 or anti-PD-L1 peptides [96]. Further experiments revealed that FGD5-AS1 can facilitate the expression of PD-L1 through miR-454-3p/ZEB1 signaling axis or miR-142-5p to promote tumor progression and cisplatin resistance [21,96], and the above mechanisms have also been verified in OC cells [56]. In addition, it has been reported that TGF-β, as one of the most important immunosuppressive cytokines secreted by M2 cells, can promote tumor metastasis and chemotherapy resistance by inducing the expression of PD-L1 [97], and secretion of TGF-β and up-regulation of PD-L1 are key factors for tumor immune escape and progress [98]. Therefore, considering that FGD5-AS1 can both activate the TGF-β signaling pathway and improve the expression of PD-L1, it should be considered as an important therapeutic target in cancer treatment.

## Future perspectives

At present, advancements in research technology have provided additional possibilities for research on lncRNAs, and scholars have focused on illustrating how specific lncRNAs are involved in the regulation of disease progression. Although lncRNAs do not encode proteins, they can regulate the expression of downstream target proteins through a variety of mechanisms. Especially for cancers, dysregulation of lncRNAs is associated with various malignant phenotypes and lead to cancer progression and metastasis. Therefore, developing new diagnostic methods and targeting inhibitors for lncRNAs may be a new approach to the fight against cancer.

LncRNA-FGD5-AS1 is a newly discovered lncRNA that has attracted considerable from researchers. Abnormally high expression of FGD5-AS1 has been reported in lung cancer [21,39–42], liver cancer [34,35], cervical cancer [53], osteosarcoma [69,70], glioma cancer [61–64], colorectal cancer [22,37], oral cancer [28,29], ovarian cancer [55,56], breast cancer [23,45], gastric cancer [30,31], pancreatic cancer [73], melanoma [72], and laryngeal squamous cell carcinoma [71] and is associated with poor prognosis in these cancers. However, several studies have also reported that FGD5-AS1 inhibits cancer. Even for the same cancer, the role of FGD5-AS1 is contradictory [30,32], which may contribute to the heterogeneity of tumor cells and tissues and different experimental methods. In terms of mechanism, FGD5-AS1 is mainly involved in the regulation of cancer progression by regulating complex ceRNA networks. For instance, FGD5-AS1 promotes the PD-L1 expression and cisplatin resistance in lung cancer cells by sponging miR-142-5p [21]. In addition, FGD5-AS1 also plays an important role in regulating the ERK/AKT signaling pathway, TGF-β signaling pathway, and Wnt/β-catenin signaling pathway. In summary, we demonstrated that FGD5-AS1 is linked to cancer and has the potential to become a therapeutic target in clinical tumor treatments.

Although many studies have revealed the relationship between FGD5-AS1 and cancer, a number of problems must be resolved before FGD5-AS1 can be applied to clinical treatment. First, the above studies are mainly at the basic research stage, and more clinical application studies need to be implemented in the future. Second, the molecular structure and specific functional regions of FGD5-AS1 are still uncertain, which is not conducive to the development of targeted drugs. CRISPR/Cas9 gene knockout, knock-in, and point mutation technologies may help us further understand the biological function of FGD5-AS1. Finally, drugs have not been tested or supported for FGD5-AS1 to date. Therefore, additional research is required on the application of FGD5-AS1 in clinical targeted therapy, tumor prognosis prediction, and chemotherapy resistance in the future.

## Data Availability

All data generated or analyzed during this study are included in the article.
